# Exploring Social Support Networks and Interactions of Young Adult and LGBTQIA+ Cancer Survivors and Care Partners

**DOI:** 10.3389/fonc.2022.852267

**Published:** 2022-04-08

**Authors:** Kristin G. Cloyes, Jia-Wen Guo, Karrin E. Tennant, Rachael McCormick, Kelly J. Mansfield, Sarah E. Wawrzynski, Sarah C. Classen, Eric C. Jones, Maija Reblin

**Affiliations:** ^1^ College of Nursing, University of Utah, Salt Lake City, UT, United States; ^2^ Department of Health Outcomes and Behavior, Moffitt Cancer Center, Tampa, FL, United States; ^3^ Department of Epidemiology, Human Genetics and Environmental Sciences, School of Public Health, University of Texas Health Science Center at Houston, El Paso, TX, United States; ^4^ Larner College of Medicine, University of Vermont, Burlington, VT, United States

**Keywords:** LGBTQIA+, cancer survivor, care partner, young adult, sexual and gender minority, social network, social support

## Abstract

**Purpose:**

The purpose of this study was to describe the social support networks and daily support interactions of cancer-affected individuals, including young adult (YA) and LGBTQIA+ survivors and care partners.

**Methods:**

Participants were recruited at two United States cancer centers and *via* social media for a pilot study testing a novel online method for collecting prospective, daily social support interaction data (N=28). All participants were aged 18+; survivors had a current or recent cancer diagnosis and were engaged in treatment and/or services; care partners were identified by the survivors. Enrollment also purposefully targeted YA and LGBTQIA+ survivors. Social network data (up to 10 members) were assessed at baseline. Daily online surveys assessed support interactions between participants and specific network members over 14 days. Descriptive statistics summarized data and explored between-group (YA/non-YA, LGBTQIA+/non-LGBTQIA+) differences in social network characteristics (size, heterogeneity, density, centralization, cohesion) and support interactions (support source and type).

**Results:**

There were no significant differences between YA and non-YA participants on any measures. LGBTQIA+ participants’ support networks were less dense (Mdn=0.69 vs. 0.82, p=.02), less cohesive (Mdn=0.85 vs. 0.91,.02), more centered on the participant (Mdn=0.40 vs. 0.24, p=.047), and included more LGBTQIA+ members (Mdn=0.35 vs. 0.00, p<.001). LGBTQIA+ participants reported having more interactions with LGBTQIA+ network members (Mdn=14.0 vs. Mdn=0.00, p<.001) and received significantly more of all types of support from LGBTQIA+ vs. non-LGBTQIA+ members. LGBTQIA+ participants also reported receiving more appraisal support than non-LGBTQIA+ (Mdn 21.64 vs. 9.12, p=.008) including more appraisal support from relatives (Mdn=11.73 vs 6.0, p+.037).

**Conclusions:**

Important information related to support access, engagement, and needs is embedded within the everyday contexts of the social networks of cancer-affected people. Individualized, accessible, and prospective assessment could help illuminate how their “real world” support systems are working and identify specific strengths and unmet needs. These insights would inform the development of more culturally competent and tailored interventions to help people understand and leverage their unique support systems. This is particularly critical for groups like YA and LGBTQIA+ survivors and care partners that are underserved by formal support services and underrepresented in cancer, caregiving, and social support research.

## Introduction

Social support, a social determinant of health that influences a range of outcomes (1), is a critical resource for people affected by cancer, including both cancer survivors and their care partners (2–4). Research has sought to explain how social support influences individuals’ cognitive and emotional appraisal of stress and, thereby, their psychosocial and physical health outcomes (5–7). Disparities in social support among diverse groups are also being more closely examined to determine how lack of access to culturally competent, relevant, and inclusive formal services and resources contributes to inequitable cancer outcomes (8–10).

Young adult (YA, aged 18-39) (11–13) and LGBTQIA+ (14–16) individuals are members of two underserved, yet growing subgroups within the cancer-affected population (17), and these groups will increasingly intersect as adolescents and YAs identify as LGBTQIA+ at higher rates than previous generations (18, 19). Younger demographics in the US also continue to grow more racially and ethnically diverse (20). Racial and ethnic minority cancer survivors in both YA and LGBTQIA+ groups experience even greater disparities in all-cause mortality, health outcomes, mental health, and quality of life (21–23).

Both YA and LGBTQIA+ groups also share characteristics that impact access to and engagement with formal support services such as lower income and financial stress, inadequacy of insurance, less traditional family and kinship systems, and lack of access to culturally competent care, increasing risk for unmet support needs. YA and LGBTQIA+ survivors have also reported feeling excluded from typical formal support services that have been largely developed with older, heterosexual, and cisgender patients living within traditional spousal relationships centered in nuclear, biological family structures; these services are not seen as relevant to their relationships and experiences (24, 25). YA and LGBTQIA+ survivors and care partners may therefore be even more reliant on informal sources of support, which may or may not be adequate to meet their needs, but this possibility has not been widely explored within either group.

Cancer-affected individuals, particularly those in underserved groups, rely on their informal social systems for support (4, 26). In the everyday lives of survivors and care partners, social support is accessed and activated within the real-time contexts of their actual personal networks through relationships and interactions that vary daily and over weeks, months and years (27). Social support networks are unique to each individual, and often include a mix of people who provide varying types of informal and formal support at different times (24). And while for many, support networks center on biological and legal relationships within nuclear family structures, this is changing as more YAs forgo marriage and traditional family structures and adopt more flexible kinship systems (28). The concept of chosen family, defined as kinship bonds formed outside of bio-legal family structures, has long been an important facet of LGBTQIA+ community (29).

Relatively little research, however, has examined the social support networks of YA and LGBTQIA+ cancer-affected individuals (30, 31). YAs rely on a mix of family, friends, and cancer peers for social support, and receive differing types of support from these sources depending on their changing needs over time and situation (32). LGBTQIA+ cancer survivors also receive support from diverse members of their networks and chosen family, which often includes friends and other LGBTQIA+ people, and they may be more likely to identify a close friend as a primary care partner (33, 34). Diverse social networks are associated with better aging and health outcomes and help buffer the stress of homophobia and transphobia (35), yet the social networks of older LGB adults may be less diverse and more tenuous than non-LGB peers (36, 37). The very few studies addressing the social support networks of transgender and gender diverse people also highlight the protective effects of adequate social support networks for buffering effects of discrimination and stress and improving health outcomes (38, 39)

The purpose of our study was to pilot an individualized, prospective, observational approach to assess characteristics of the personal social support networks and patterns within daily support interactions of a sample of cancer survivors and care partners focusing on YA and LGBTQIA+ individuals. To do so, we developed a novel online method combining social network assessment (structural factors) and prospective daily interaction diaries (functional factors) and we report on the development, feasibility, and acceptability of these methods elsewhere (40). Here we present the results of our descriptive analysis of participants’ personal social support networks and interactions with network members which included exploratory between-group comparisons (YA/non-YA and LGBTQIA+-non-LGBTQIA+.).

## Methods

### Study Design

We employed a prospective cohort design. All study activities were reviewed and approved by institutional review boards for the protection of human subjects at both study sites. This study was determined to be exempt by the University of Utah IRB (#00119352) and the Advarra IRB (Moffitt Cancer Center; Review #MCC20021).

### Setting and Recruitment

A purposive, non-random sample of participants meeting study eligibility criteria were recruited from populations served by two comprehensive cancer centers in the Intermountain West and the Southeast regions of the United States and nationally *via* community partners’ social media channels. Rolling recruitment occurred between August 2019 and May 2020. As our primary aim was to pilot test the feasibility of our methods within hard-to-reach populations (i.e. YA, LGBTQIA, care partners) and provide proof of concept for prospective assessment of complex social network and support data, the small sample size and use of purposive sampling methods were acceptable strategies (41).

### Participants

All eligible participants were 18 and older, able to speak and read English, and were either a cancer survivor (broadly defined according to the NCI definition of a person who is on a trajectory from cancer diagnosis to end of life) (42) or a care partner of a cancer survivor (a person who most often helps the survivor and is not paid to do so). Eligible survivors had at least one current or historical cancer diagnosis, were currently engaged in treatment, services, monitoring, or follow-up related to this diagnosis, and were able to identify a primary, informal care partner or support person who also consented to participate in the study. Additionally, they had to be either YA or self-identify as LGBTQIA+ or both. Eligibility criteria for care partners included a person who the survivor considers a main source of routine support who also consented to participate. We had originally intended to enroll eight YA survivor/care partner dyads and eight LGBTQIA+ survivor/care partner dyads for a target enrollment of N=32. This was complicated by the onset of the COVID-19 pandemic in the US, however, and we stopped enrollment before fully accruing as planned. Each participant was screened by research staff for inclusion criteria, participated in the informed consent process, and was compensated $100 on study completion.

### Measures

At baseline, participants completed a demographic survey in REDCap (43) and an interview-based assessment in which ecomaps were constructed to assess characteristics of their personal (egocentric) social support networks. Interview responses and ecomaps were recorded and transferred by research staff into REDCap. Participants’ network member data were then used to personalize a daily electronic survey that was texted or emailed to participants for 14 consecutive days. These prospective daily surveys assessed characteristics of participants’ daily interactions with network members, described below. While participants included survivor and care partner dyads, individual participant data were not shared by study team members.

#### Demographics

Demographic data included cancer-related role (survivor or care partner), age, racial identity, Hispanic/Latinx ethnicity, sexual orientation, gender, cis- or transgender status, relationship status, highest level of education, and income.

#### Social Network Measures

Each participant completed an individual baseline interview with a researcher in which an eco- mapping technique was used to elicit egocentric social network information. Participants were asked to identify up to 10 people in their social network they considered to be important sources of support related to their cancer experience (e.g. spouses/partners, relatives, friends, neighbors, co-workers, spiritual advisors, case managers, therapists). For each person, participants provided the following data: First name or initials, age, gender, whether the member was LGBTQIA+, the participants’ primary relationship to the network member, length of time known, closeness of the relationship between the participant and each network member, and whether/how specific network members were connected with other network members.

#### Daily Interaction Surveys

Based on participant preference, first names or initials of network members were then incorporated into brief daily REDCap surveys assessing characteristics of participants’ interactions with the identified members (alters) of their support network. Each day for 14 days, participants received a link *via* text or email to an online survey presenting a list of their network members and were asked to select which members they had interacted with during the last 24 hours. For each network member selected, participants were then asked to focus on one interaction with that member during the past 24 hours and provide the following information about that focal interaction: the purpose of the interaction (free text response), whether the interaction was perceived as supportive (yes, no, not meant to be supportive), the type of support the interaction represented for the participant (instrumental, informational, emotional, appraisal, based on definitions and examples provided for participants), and perception of helpfulness of the interaction (5-point Likert rating, 0 = not at all helpful, 5 = very much helpful). Only interactions perceived as supportive (yes vs. no/not mean to be) and rated as at least somewhat helpful (≥ 2 Likert rating) were included in analysis.

### Analysis

Study data from both sites were merged, and all study data were reviewed and checked for consistency and errors. Missing data analysis was conducted to assess the pattern of missingness for the baseline psychosocial measures; multiple imputation was used to impute missing data after missing completely at random was confirmed. Descriptive statistics were used to summarize baseline and daily interaction data using both SPSS (version 27) and R software. UCINET (44) software was used to calculate social network variables for density (the extent to which most or all participants’ network members know each other), degree centralization (the extent to which connections within one’s network are numerically dominated by one or few individuals, including the participant), and cohesion (the extent to which the network is more connected vs. disconnected, somewhat irrespective of density). Three network heterogeneity measures were also calculated: diversity of age of network members, the ratio of relatives to non-relatives, and the ratio of LGBTQIA+ to non-LGBTQIA+ members. Because of the small sample size and the nonnormality of psychosocial, network, and daily interaction data, Mann-Whitney U tests were used to compare between-group differences (i.e., YA vs. non-YA, LGBTQIA+, vs. non-LGBTQIA+). A significance level of p = 0.05 was set for all tests, and we report exact p values where possible.

## Results

### Participant Demographics

**Table 1** presents participant demographics. Most participants were White (n=24, 86%), not Hispanic/Latinx (n=23, 96%), female (n=19, 67.9%), and cisgender (n=25, 89%). Seventeen participants were heterosexual (61%), and 11 were either lesbian, gay, bisexual, queer, or pansexual (39.3%). There were no significant between-group differences for demographics aside from non-heterosexual and transgender and nonbinary categories.

**Table 1:** Participant demographics.

	All (N = 28)	LGBTQIA+ (n = 11)	Non-LGBTQIA+ (n = 17)
	Mean Yrs (SD)		
**Age**	40.75 (18.26)	39.64 (22.23)	41.47 (15.89)
**Cancer Role**	N (%)		
Survivor	14 (50)	6 (54.54)	8 (47.06)
Care Partner	14 (50)	5 (45.45)	9 (52.94)
**Gender**			
Man	8 (28.57)	3 (27.27)	5 (29.41)
Woman	19 (67.86)	7 (63.64)	12 (70.59)
Non-Binary	1 (3.57)	1 (9.09)	--
**Trans/Cisgender**			
Transgender	3 (10.71)	3 (27.27)	--
Cisgender	25 (89.29)	8 (72.72)	17 (100)
**Sexual Orientation**			
Heterosexual	17 (60.71)	--	17 (100)
Lesbian/Gay	3 (10.71)	3 (27.27)	--
Bisexual	4 (14.29)	4 (36.36)	--
Queer	2 (7.14)	2 (18.18)	--
Pansexual	2 (7.14)	2 (18.18)	--
**Race**			
Black	4 (14.29)	--	4 (23.53)
White	24 (85.71)	11 (100)	13 (76.47)
**Ethnicity**			
Latinx	1 (3.57)	1 (9.09)	--
**Relationship Status**			
Single (Never married)	3 (10.71)	--	3 (17.65)
Separated or Divorced	1 (3.57)	--	1 (5.88)
Married	15 (53.57)	4 (36.37)	11(64.71)
Registered domestic Partnership or Civil union	--	--	--
Committed relationship (not legally or officially married or registered)	7 (25)	7 (63.63)	--
Widowed	2 (7.14)	--	2 (11.77)
**Education**			
High school	3 (10.71)	--	3 (17.64)
Some college or vocational school	9 (32.14)	3 (27.27)	6 (35.29)
College Graduate	1 (3.57)	1 (9.09)	--
Some graduate or professional schooling	4 (14.29)	3 (27.27)	1(5.88)
Graduate or professional degree	11 (39.29)	4 (36.36)	7 (41.18)
**Income**			
Less than $9,999	2 (7.14)	1 (9.09)	1 (5.88)
$10,000-$24,999	6 (21.43)	4 (36.37)	2 (11.77)
$25,000-$39,999	3 (10.71)	1 (9.09)	2 (11.77)
$40,000-$49,999	1 (3.57)	--	1 (5.88)
$50,000-$74,999	--	--	--
$75,000-$99,999	1 (3.57)	1 (9.09)	--
>$100,000	11 (39.29)	3 (27.27)	8 (47.05)
Prefer not to disclose	4 (14.29)	1 (9.09)	3 (17.65)

### Social Network Characteristics

Participants’ support networks had a mean of six members (SD = 2.22, range 2-10) and represented a mix of relatives (e.g. sibling, in-law; M = 3.6, SD = 1.4, range 1-7) and non-relatives (e.g. friend, co-worker; M = 2.8, SD = 2.3, range 1-7). Between-group comparisons showed no significant differences in network size/number of network members.

There were no significant differences between YA and non-YA support networks composition or structure. **Table 2** presents a comparison of LGBTQIA+ and non-LGBTQIA+ social network characteristics. The support networks of LGBTQIA+ participants were significantly less dense (Mdn = 0.69 vs. 0.82, p = .02), less cohesive (Mdn = 0.85 vs. 0.91, p = .02) and more degree centralized (Mdn = 0.4 vs. 0.24, p = .047) than those of non-LGBTQIA+ participants. They also were significantly more heterogeneous in terms of having more LGBTQIA+ members than the networks of non-LGBTQIA+ participants (Mdn = 0.35 vs. 0.00, p <.001). While not significantly different, the support networks of LGBTQIA+ participants also tended to be more heterogenous for member age (Mdn = 15.89 vs. 14.7).

**Table 2:** Social network characteristics.

Variables	LGBTQIA+n = 11			Non-LGBTQIA+n = 17			MW	
	M	SD	Mdn	M	SD	Mdn	U	p
Number of ties (network size)	6.82	1.99	7.0	5.47	2.18	5.0	59.00	0.1
Heterogeneity-Relatives/ Non-relatives	0.34	0.18	0.38	0.31	0.21	0.38	85.50	0.71
Heterogeneity-Age	14.9	5.15	15.89	6.9	27.64	14.17	69.00	.26
Heterogeneity-SGM/ Non-SGM	0.33	0.11	0.35	0.08	.15	0.00	22.00	<.001*
Density	0.68	0.15	0.69	.834	0.16	0.82	44.50	.02*
Centralization	0.42	0.19	0.40	0.24	0.06	0.24	51.50	.047*
Cohesion	0.84	0.08	0.85	0.92	0.08	0.91	45.00	.02*

MW, Mann-Whitney U test; M, mean; SD, standard deviation; Mdn, median; *Significant at p < 0.05 level.

### Daily Interactions and Perceived Support

We examined the total number of reported support interactions overall and with unique network members for each participant, focusing on relationship type (relatives vs. non-relatives), LGBTQIA+ status (LGBTQIA+ vs. non-LGBTQIA+ network members), and on the number of support interactions for each type of perceived support (emotional, informational, appraisal, instrumental).

Participants reported a mean of 41.79 support interactions overall during the two-week period (Mdn=39, SD=26.3, range=8-108) and a mean of 27.8 interactions with different network members (M= 27.75, Mdn=25.5, SD=15.6, range=4 - 58). There were no significant differences between YAs and non-YAs for number of interactions of any specific support type (instrumental, informational, emotional, appraisal, other) overall, or when examining support type by source (relative or non-relative.)

While not statistically significant, LGBTQIA+ participants reported more daily interactions with non-relatives than with relatives overall (**Table 3**, p = .08). LGBTQIA+ participants reported more appraisal support interactions with all members in their network (relatives and non-relatives) compared to non-LGBTQIA+ participants (p = .008), including more appraisal support interactions with relatives (p = .037).

**Table 3:** LGBTQIA+ and non-LGBTQIA+ support interactions with relatives and non-relatives.

Variables	LGBTQIA+n = 11			Non-LGBTQIA+n = 17			MW	
	M	SD	Mdn	M	SD	Mdn	U	p
Count of **daily interactions** with:								
Relatives	18.00	7.96	16.00	19.47	12.53	18.00	92.50	.96
Non-relatives	14.45	12.74	12.00	5.59	6.78	4.00	131.00	.08
All members	32.36	17.48	26.00	24.76	14.01	24.00	114.00	.35
Count of **emotional** support interactions with:								
Relatives	10.09	7.62	10.00	7.41	7.67	4.00	119.50	.23
Non-relatives	7.27	9.82	5.00	2.71	5.19	1.00	125.50	.13
All members	17.36	14.31	14.00	10.12	11.5	5.00	132.00	.07
Count of **informational** support interactions with:								
Relatives	3.45	3.21	3.00	6.18	7.69	4.00	80.00	.55
Non-relatives	2.09	2.63	1.00	2.00	3.48	1.00	98.00	.85
All members	5.55	5.3	4.00	8.18	9.02	4.00	85.00	.71
Count of **appraisal** support interactions with:								
Relatives	11.73	8.00	12.00	6.00	7.42	3.00	138.00	.037*
Non-relatives	9.91	10.77	7.00	3.12	4.46	0.00	134.00	.06
All members	21.64	13.69	15.00	9.12	9.33	6.00	149.00	.008*
Count of **instrumental** support interactions with:								
Relatives	8.09	6.09	9.00	6.53	6.98	3.00	111.00	.43
Non-relatives	3.55	7.09	0.00	0.71	1.53	0.00	118.00	.26
All members	11.64	7.67	12.00	7.24	7.12	7.00	130.50	.08

MW, Mann-Whitney U test; M, mean; SD, standard deviation; Mdn, median; *Significant at p < 0.05 level.

LGBTQIA+ participants reported more interactions with LGBTQIA+ members than with non-LGBTQIA+ members (**Table 4**, p <.001), and more emotional support (p <.001), appraisal support (p <.001), and instrumental support from LGBTQIA+ vs. non-LGBTQIA+ members (p <.001). LGBTQIA+ participants also reported more informational interactions with both LGBTQIA+ (p = .006) and non-LGBTQIA+ (p = .019) alters, and more appraisal support from all members (p = .008), than did non-LGBTQIA+ participants.

**Table 4:** Support interactions with LGBTQIA+ and non-LGBTQIA+ network members.

Variables	LGBTQIA+n = 11			Non-LGBTQIA+n = 17			MW	
	M	SD	Mdn	M	SD	Mdn	U	p
Count of **daily interactions** with:								
LGBTQIA+ members	15.64	10.74	14.00	1.65	3.64	0.00	179.50	<.001*
Non-LGBTQIA+ members	16.55	14.72	12.00	25.88	14.33	26.00	58.00	.10
All members	32.64	17.06	26.00	27.53	14.93	29.00	108.00	.52
Count of **emotional** support interactions with:								
LGBTQIA+ members	11.00	6.93	10.00	0.47	1.23	0.00	176.50	<.001*
Non-LGBTQIA+ members	5.91	11.30	2.00	10.12	11.30	6.00	53.00	.06
All members	17.36	14.31	14.00	10.59	11.41	6.00	128.50	.10
Count of **informational** support interactions with:								
LGBTQIA+ members	3.27	3.04	3.00	0.41	1.00	0.00	150.50	.006*
Non-LGBTQIA+ members	1.82	2.04	2.00	7.94	9.02	5.00	44.00	.019
All members	5.55	5.30	4.00	8.35	8.91	5.00	80.50	.55
Count of **appraisal** support interactions with:								
LGBTQIA+ members	11.64	7.66	12.00	0.47	1.13	0.00	177.00	<.001*
Non-LGBTQIA+ members	9.64	10.99	5.00	9.06	9.24	5.00	95.00	.96
All members	21.64	13.69	15.00	9.53	9.26	7.00	149.00	.008
Count of **instrumental** support interactions with:								
LGBTQIA+ members	7.91	6.02	8.00	0.18	0.73	0.00	176.00	<.001*
Non-LGBTQIA+ members	3.36	3.30	2.00	7.06	7.08	7.00	67.50	.23
All members	11.64	7.67	12.00	7.24	7.12	7.00	130.50	.08

MW, Mann-Whitney U test; M, mean; SD, standard deviation; Mdn, median; *Significant at p < 0.05 level.

## Discussion

For an increasingly diverse population of cancer-affected people, there remain challenges to assessment and inclusion in survivorship, social support, and social network research that may limit the impact of this knowledge and its implementation in real-world contexts (45). Despite how many cancer survivors rely on informal support systems, little social support research accounts for the unique social context of the individual—this is a critical gap in achieving equity for groups whose support systems may look or work differently from the norms that currently inform assessment and intervention. Many of the influential findings related to social support in cancer and caregiving populations draw from research conducted with mostly white, heterosexual, cisgender, middle-aged, and older adults (8, 46). And while dyadic social support and cancer research expands beyond the individual perspective, it still largely ignores the possibility that other kinds of social relationships may be just as critical to supporting survivors and care partners as traditional spousal and kin relationships (47). Existing research also ignores the reality that for many groups, social support and caregiving are not centered in spouse/partner or biological parent/child dyads, but distributed across social networks comprising an array of members who may be more or less demographically and developmentally diverse (48). This pilot study uses novel methodology to address gaps in previous research and assess structural and functional aspects of personal social support networks of underserved YA and LGBTQIA+ cancer patients.

Though our analyses revealed no significant differences between social network characteristics of YA and non-YA participants, contrary to expectation (49), we did note significant differences in network characteristics based on LGBTQIA+ status (50, 51). LGBTQIA+ participants’ networks were less dense and less cohesive, suggesting that LGBTQIA+ participants’ connections to social support may be more diffused across network members. The higher degree centralization of LGBTQIA+ participants in their social networks (i.e., members are generally connected to the participant but less with each other) suggests that these individuals were playing a more central role in holding their own networks together. LGBTQIA+ participants’ networks were also more diverse in that they included more LGBTQIA+ members, plus LGBTQIA+ participants also had more support-related interactions overall, including more with non-relatives.

These network characteristics may be strengths, offering participants a wide reach of network members with frequent contact (52, 53). Diffuse and heterogeneous networks have been shown to have benefits, including brokering diverse information and access to an array of resources (54). Further, higher levels of perceived support may mitigate negative health effects related to less cohesive and more diffuse networks (55, 56). However, a combination of higher heterogeneity and degree centralization with less density and cohesion may also be associated with a diffusion of social support and overall weaker connections among network relationships, which may also put LGBTQIA+ participants in a more precarious position in more volatile and high need situations, such as when participants are ill or burdened. For example, if a support network is dependent on a cancer survivor or care partner as a stabilizing node of connection, with few strong, well-resourced ties, their inability to fulfill the connecting role due to illness, lack of resources to mobilize the network, or competing demands can lead to a lack of coordinated support. This network profile may create problems for accessing or leveraging social support resources and could lead to unmet support needs. This premise should also be tested in a larger study over a longer period of time.

Emotional and appraisal support appeared to be an important type of support for LGBTQIA+ participants, as they reported more of these interactions with all network members compared with non-LGBTQIA+ participants. Appraisal support provides individuals with a sense of context that supports self-evaluation, reflection on one’s situation and standing, and a sense of connection to others who know and understand you well enough to afford this perspective (57). For individuals who are also LQBTQIA+ who experience minority stress–a combination of proximal and distal stressors related to minority status that span intrapersonal, interpersonal, and structural factors, the role of appraisal-oriented support may be particularly relevant to perceptions of support and mental health outcomes (58). LGBTQIA+ individuals, including younger people, are likely to have more experience with minority stress than are their non-minority counterparts and may have more practice and facility with accessing appraisal and emotional support within their networks, which may be protective. The flip side of this dynamic, however, is the compounded risk associated with LGBTQIA+ cancer survivors and care partners not having stable connections to people who provide this support within their personal networks and/or not knowing how to ask for and generate this support among members.

### Implications for Intervention

Future work should examine how formal sources of support (e.g. oncologists, therapists, counselors) are integrated within survivors’ and care partners’ existing social networks (or not), including interactions between timing and types of support, service use, and wellbeing. The social support systems of cancer-affected people are inherently unique and—for YAs and people in minoritized populations like LGBTQIA+–may not be well-reflected in the expectations and operations of established formal services. For example, routine clinical care may hold implicit expectations of a hetero-normative network more traditionally seen in research on older adults, in which a spouse or adult child is available to provide outpatient care to patients. Those whose networks do not conform to this standard may not only experience feelings of alienation, but may be missing critical support for their care.

Developing a working understanding of more diverse social support landscapes is therefore important for clinicians as well as researchers. Clinicians can be mindful of expectations for support required for patients and caregivers to participate effectively in treatment and facilitate connections to inclusive formal resources when additional support is needed. Across healthcare systems, more tailored, less generic patient-centered clinical and support services would be particularly impactful for groups like YA and LGBTQIA+ who report feeling alienated by normative care models that do not acknowledge their individual contexts and experiences.

Researchers should focus on developing personalized interventions that boost cancer survivors’ and cancer care partners’ self-efficacy in accessing and leveraging social support to meet everyday challenges. This is especially critical for underserved populations, including people who are YA and LGBTQIA+ cancer survivors or care partners, who may be even more reliant on their personal social networks who feel disconnected from typical, formal support resources, and whose support systems may look different than those represented in the cancer literature. Finding ways to better coordinate holistic, high-quality cancer care is a national priority (59). Conducting more inclusive survivorship research will be important to ensure health care policy remedies, rather than reinforces, health care disparities.

### Limitations

There were a number of limitations to this study. The descriptive and exploratory nature of our analyses limits the generalizability of our findings. A small sample size and purposive, non-probability sampling may have increased the risk of selection bias. There was a wide age range within the LGBTQIA+ group which may be contributing to the differences we found, although we verified that groups did not significantly differ by age. While grouping individuals of diverse sexual orientations and gender identities together in a single LGBTQIA+ group is often necessary due to small numbers of participants in these groups, it confounds important between-group differences that are relevant to understanding cancer- and minority-stress related support needs; this is compounded by the lack of representation of transgender and gender diverse participants in most studies, including this one. Finally, our data collection period spanned August 2019-May 2020 and the onset of the COVID-19 pandemic in the US slowed our study enrollment considerably, leading us to close recruitment before enrolling an equal number of LGBTQIA+ and non-LGBTQIA+ participants.

### Conclusion

Every cancer-affected individual’s social support system is unique. Both structural and functional aspects of social support networks–network characteristics and patterns of interactions within these networks—are likely to influence survivors’ perceptions of support, appraisal of stress, capacity to cope, and ultimately their well-being. These influences may differ from person to person and by groups affected by differing social determinants of health (60). Relationship types and quality, the closeness of member connections, modality of interactions, patterns of interaction over time, and survivors’ changing preferences, needs, and perceptions of helpfulness are also likely to shape how social support affects survivors’ appraisal and coping (61). Examining these complexities for diverse groups of cancer survivors and their care partners should, therefore, be a priority for developing and implementing culturally-relevant interventions.

We sought to examine the personal social support networks of cancer survivors and their care partners in two groups—YA and LGBTQIA+ cancer survivors—that have been under-represented in survivorship, caregiving, and social support research and who subsequently report unmet support needs. We further sought to contextualize this by studying the survivor-care partner dyads in relation to their personal support networks. This study provides proof of concept for this strategy, and suggests that there may be important aspects of YA and LGBTQIA+ survivor cohorts in the structure and function of their personal support networks. Next steps include adapting all data collection methods for online access, repeating the protocol with a larger sample over a longer period of time, and modeling how social network characteristics and daily interaction patterns predict changes in perceived stress, support, and mental health outcomes.

## Data Availability Statement

A limited de-identified data set is available on request. Requests to access the datasets should be directed to kristin.cloyes@nurs.utah.edu.

## Ethics Statement

The studies involving human participants were reviewed and approved by University of Utah IRB, and the Advarra IRB Moffitt Cancer Center. The patients/participants provided their written informed consent to participate in this study.

## Author Contributions

KGC and MR conceptualized this study and acquired project funding. KGC developed the methodology for data collection. KET, REM, and KJM coordinated project administration. J-WG developed the plan for formal analysis. KGC, J-WG, EJC, SCC, and SEW performed data analysis. KGC wrote the original draft. All authors contributed to the article and approved the submitted version.

## Funding

This study was supported by a Dick and Timmy Burton Foundation Pilot Grant Award, University of Utah College of Nursing, Moffitt Cancer Center Adolescent and Young Adult Program with support from Swim Across America and the Bay Area Advisors, K01NR016948 (J-WG’s effort), T32NR013456 (KJM’s effort), NINR F31NR018987 (SEW’s effort). The REDCap application reported in this publication was supported in part by the National Center for Advancing Translational Sciences of the National Institutes of Health under Award Number UL1TR001067 for period 5/1/13–3/30/18 and UL1TR002538 for period 4/1/18-2/28/23.

## Conflict of Interest

The authors declare that the research was conducted in the absence of any commercial or financial relationships that could be construed as a potential conflict of interest.

## Publisher’s Note

All claims expressed in this article are solely those of the authors and do not necessarily represent those of their affiliated organizations, or those of the publisher, the editors and the reviewers. Any product that may be evaluated in this article, or claim that may be made by its manufacturer, is not guaranteed or endorsed by the publisher.
